# Quantification of epicardial adipose tissue in obese patients using an open-bore MR scanner

**DOI:** 10.1186/s41747-022-00274-0

**Published:** 2022-05-24

**Authors:** Francesco Secchi, Carmela Asteria, Caterina B. Monti, Alexis Elias Malavazos, Davide Capra, Marco Alì, Cecilia L. A. Giassi, Simona Francesconi, Sara Basilico, Alessandro Giovanelli, Lelio Morricone, Francesco Sardanelli

**Affiliations:** 1 Department of Biomedical Sciences for Health, Università degli Studi di Milano, Via Mangiagalli 31, 20133 Milan, Italy; 2 Department of Radiology, IRCCS Policlinico San Donato, San Donato Milanese, Italy; 3 National Institute for Obesity Cure (INCO), IRCCS Policlinico San Donato, San Donato Milanese, Italy; 4 Endocrinology Unit, Clinical Nutrition and Cardiovascular Prevention Service, IRCCS Policlinico San Donato, San Donato Milanese, Italy; 5 Department of Biomedical, Surgical and Dental Sciences, Università degli Studi di Milano, Milan, Italy; 6 Unit of Diagnostic Imaging and Stereotactic Radiosurgery, C.D.I. Centro Diagnostico Italiano S.p.A., Milan, Italy; 7 Bracco Imaging S.p.A., Via Caduti di Marcinelle 13, 20134 Milan, Italy

**Keywords:** Magnetic resonance imaging, Reproducibility of results, Heart, Adipose tissue, Bariatric surgery

## Abstract

**Background:**

Our aim was to evaluate the reproducibility of epicardial adipose tissue (EAT) volume, measured on scans performed using an open-bore magnetic resonance scanner.

**Methods:**

Consecutive patients referred for bariatric surgery, aged between 18 and 65 years who agreed to undergo cardiac imaging (MRI), were prospectively enrolled. All those with cardiac pathology or contraindications to MRI were excluded. MRI was performed on a 1.0-T open-bore scanner, and EAT was segmented on all scans at both systolic and diastolic phase by two independent readers (R1 with four years of experience and R2 with one year). Data were reported as median and interquartile range; agreement and differences were appraised with Bland-Altman analyses and Wilcoxon tests, respectively.

**Results:**

Fourteen patients, 11 females (79%) aged 44 (41–50) years, underwent cardiac MRI. For the first and second readings, respectively, EAT volume was 86 (78–95) cm^3^ and 85 (79–91) cm^3^ at systole and 82 (74–95) cm^3^ and 81 (75–94) cm^3^ at diastole for R1, and 89 (79–99) cm^3^ and 93 (84–98) cm^3^ at systole and 92 (85–103) cm^3^ and 93 (82–94) cm^3^ at diastole for R2. R1 had the best reproducibility at diastole (bias 0.3 cm^3^, standard deviation of the differences (SD) 3.3 cm^3^). R2 had the worst reproducibility at diastole (bias 3.9 cm^3^, SD 12.1 cm^3^). The only significant difference between systole and diastole was at the first reading by R1 (*p* = 0.016). The greatest bias was that of inter-reader reproducibility at diastole (-9.4 cm^3^).

**Conclusions:**

Reproducibility was within clinically acceptable limits in most instances.

## Key points

- Fourteen obese patients underwent cardiac MRI on a 1.0-T open-bore scanner.
- Two readers measured epicardial adipose tissue at systolic and diastolic phase.
- Reproducibility was within clinically acceptable limits in most instances.

## Background

Epicardial adipose tissue (EAT) is a visceral fat depot located between the myocardium and visceral epicardium, surrounding the coronary vessels [1], with thermogenic functions due to its nature as a “beige” adipose tissue [2], and numerous endocrine, paracrine and vasocrine interplays with the neighbouring structures [3]. Even though EAT physiologically produces anti-inflammatory cytokines such as adiponectin and provides antiatherogenic and cardioprotective effects, in pathological conditions, it may produce proinflammatory cytokines and promote the development of coronary artery disease (CAD) [4].

Pathological changes induce an increase in EAT volume due to inflammation [5], and such changes may be regarded as an early biomarker of CAD. The volume of EAT can be assessed via non-invasive imaging studies such as computed tomography and magnetic resonance imaging (MRI) [6, 7]. Both techniques allow to estimate the EAT volume on routine scans, without the need of contrast agent injection. In particular, MRI, which does not expose the patient to ionising radiation, allows to assess EAT on cine bright-blood, steady-state free precession images, which are a mainstay in every routine cardiac MRI examination.

Among the populations where CAD risk is higher and evaluating EAT may provide an advantage are obese patients [8]. Notably, EAT was shown to play a crucial role in the development of cardiovascular diseases in the obese population, acting as a transducer of systemic inflammation and metabolic dysregulation from the whole body to the heart [9]. However, MRI assessment of EAT in obese patients is challenging, as the bore of MRI scanners may not be sufficiently wide to accommodate large sizes [10]. A potential solution for this issue is the use of open-bore MRI scanners, which have been shown to provide at least a subjective image quality comparable to that provided by closed-bore scanners [11]. Nevertheless, data concerning the accuracy and precision of quantifying EAT using images acquired from open-bore MR scanners are still scarce.

Therefore, the purpose of our study was to evaluate intra- and inter-reader reproducibility of EAT volumes, measured on scans performed with cine sequences in a population of obese patients using an open-bore MR scanner.

## Methods

The Policlinico San Donato Research Hospital (IRCCS) promoted a multicentre observational study in collaboration with the Istituto Clinico Sant’Ambrogio, and the Centro Diagnostico Italiano aiming at the evaluation of EAT in patients undergoing bariatric surgery. The present research is a sub-study of the main project, analysing MRI examinations performed before surgical intervention.

Ethical approval was obtained for all centres involved in this study (Ethics Committee of San Raffaele Research Hospital approved the study on May 11, 2017; protocol code: EAT-BS). All subjects signed a dedicated informed consent.

### Study population

Between June and October 2017, patients referred for bariatric surgery with a body mass index ≥ 40 kg/m^2^ or ≥ 35 kg/m^2^ in the presence of comorbidities (cardiovascular, respiratory, or metabolic pathologies, severe articular pathology, or psychological comorbidities) whose age was between 18 and 65 years, and who agreed to undergo cardiac MRI before surgery, were prospectively enrolled. Exclusion criteria were the presence of overt cardiac pathology such as ischaemia or valvular disease, pregnancy or contraindications to bariatric surgery, along with the main contraindications to MRI, namely unsafe or conditional devices, intracranial ferromagnetic clips, intraocular metallic chippings, severe claustrophobia and impossibility to maintain supine position or avoid involuntary movements. Moreover, patients with heavily artifacted MR images were excluded from analysis.

### Image acquisition

All MRI examinations were performed using a 1.0-T open-bore scanner (Panorama, Philips Medical Systems, Best, The Netherlands), equipped with 26 mT/m gradient power, using either an 8-channel surface phased array coil (SENSE Body-L, Philips Medical Systems, Best, The Netherlands) or a 3-channel surface phased array coil (SENSE Body-XL, Philips Medical Systems, Best, The Netherlands), depending on the size of the patient. For each patient, an electrocardiographic-triggered cine steady-state free precession sequence was acquired, in a short axis covering the heart from the apex to the basal portion, with the following parameters: slice thickness 10 mm, field of view 320 × 100 mm^2^, flip angle 70°, time of repetition 4.7 ms and time of echo 2.1 ms.

### Image analysis

**Fig. 1:**
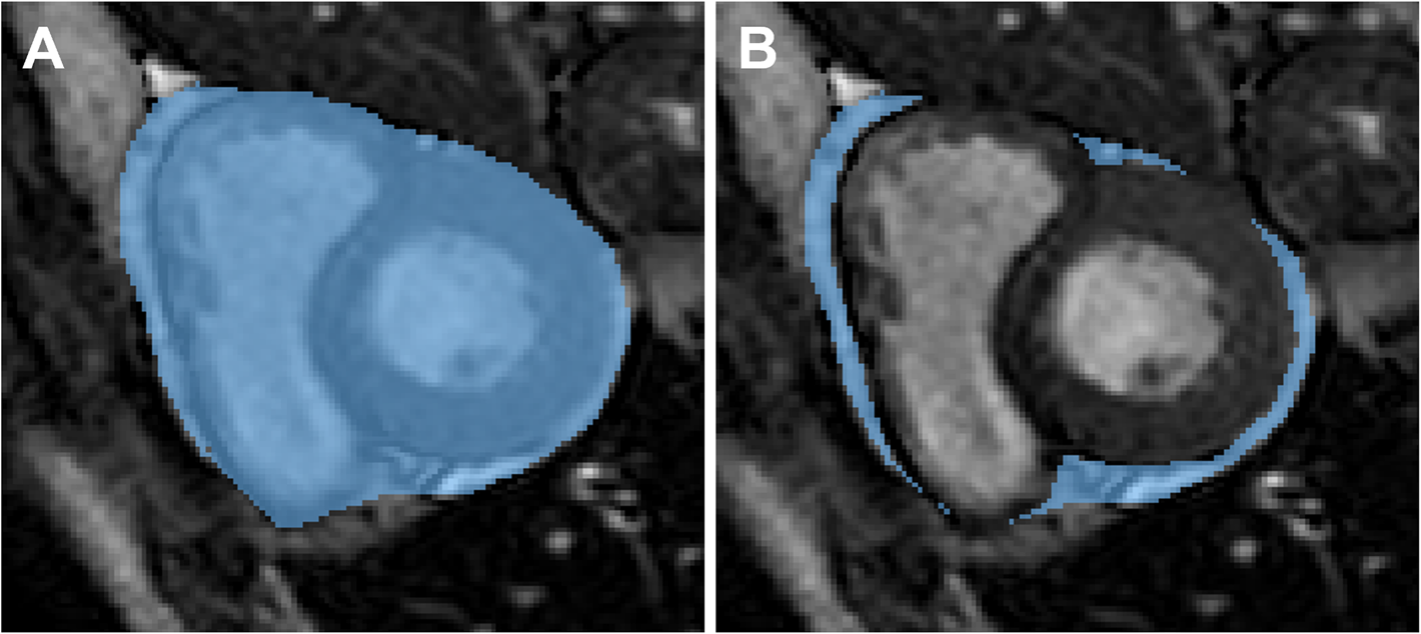
Segmentation of the pericardium (**A**) with subsequent subtraction of the epicardium (**B**) to obtain an estimate of epicardial adipose tissue volume in an image obtained from a cine sequence in short axis acquired on an open-bore MR scanner in a 44-year-old male patient referred for bariatric surgery

For each patient, the pericardium was segmented on short-axis cine images by two readers, with a four-year (R1) and one-year (R2) experience in cardiovascular MRI. Readers manually traced the contour of the pericardium on every slice, using a freeware software, ITK-SNAP Version 3.8.0 (www.itksnap.org) [12], as depicted in Fig. 1, both on systolic and diastolic frames which were previously chosen by the two readers in consensus. Afterwards, they traced the contour of the epicardium to exclude tissues different from EAT which were included in the previous segmentation, as depicted in Fig. 1. Along the longitudinal cardiac axis, the segmentation included slices from the cardiac base to its apex. We used the short axis stack as it provides the best visualisation of the pericardial sac, thus facilitating segmentation. However, during the segmentation, a multiplanar reconstruction was available to the reader, providing additional spatial information whenever needed. The EAT volume was obtained by multiplying the areas by the slice thickness slice by slice, then summing all the values to obtain measure expressed as cubic centimetres. Both readers performed all measurements twice, with a time interval between the reading sessions of at least two weeks.

### Statistical analysis

A non-normal distribution was assumed due to the paucity of data; hence, data were reported as median and interquartile range (IQR). Correlations were assessed with Bland-Altman analyses and reported as bias and standard deviation (SD). Differences were analysed with the Wilcoxon test for paired variables. Any *p*-value ≤ 0.05 was considered indicative of statistical significance [13]. Statistical analyses were performed using Python 3.7.6.

## Results

### Study population

Out of 17 initially enrolled patients, at the time of writing this report, 14 had undergone the pre-operative MRI, while the others had not due to size issues (*n* = 3), *i.e.*, exceeding the vertical diameter of the open-bore gantry. Thus, our study population was composed of 14 patients, 11 (79%) of whom were females, with a median age of 44 years (IQR 41–50 years). Details on our population characteristics are reported in Table 1.

**Table 1:** Population characteristics

	Patients (*n* = 14)
Females (*n*, %)	11 (79)
Age (years)	44 (41–50)
Weight (kg)	107 (101–112)
Height (cm)	162 (159–183)
BMI (kg/m^2^)	43.4 (40.8–45.9)

*BMI*, Body mass index. Data is reported as median and interquartile range unless otherwise stated

### Epicardial adipose tissue volume reproducibility

EAT volumes measured by R1 and R2 at different cardiac phases and readings are reported in Table 2, while reproducibility data are reported in Table 3. The median EAT volume estimates were 86 cm^3^ (IQR 78–95 cm^3^) at systole and 82 cm^3^ (IQR 74–95 cm^3^) at diastole for the first read of R1 while they were 89 cm^3^ (IQR 79–99 cm^3^) at systole and 92 cm^3^ (IQR 85–103 cm^3^) at diastole for the first read of R2.

**Table 2:** Median epicardial adipose tissue volume at systole and diastole with different readers

	EAT volume (cm^**3**^)		Wilcoxon ***p***-value
	Systole	Diastole	
**R1.1**	86 (78–95)	82 (74–95)	0.016
**R1.2**	85 (79–91)	81 (75–94)	0.124
**R2.1**	89 (79–99)	92 (85–103)	0.551
**R2.2**	93 (84–98)	93 (82–94)	0.638

Values are median and interquartile range

*R1.1*, First measurements from reader 1; *R1.2*, Second measurements from reader 1; *R2.1*, First measurements from reader 2; *R2.2*, Second measurements from reader 2

**Table 3:** Results of Bland-Altman analyses for the assessment of intra-reader, inter-reader and inter-phase (systole and diastole) reproducibility

		Reproducibility (cm^**3**^)	
		Systole	Diastole
**Intra-reader**	**R1**	Bias 1.5; SD 5.0	Bias 0.3; SD 3.3
	**R2**	Bias − 1.6; SD 7.3	Bias 3.9; SD 12.1
**Inter-reader**	**R1**–**R2**	Bias − 2.3; SD 20.6	Bias − 9.4; SD 14.9
**Inter-phase**	**R1**	Bias 3.3; SD 4.6	
	**R2**	Bias − 3.8; SD 12.1	

All reported data are in cm^3^

*R1*, reader 1; *R2*, reader 2

**Fig. 2:**
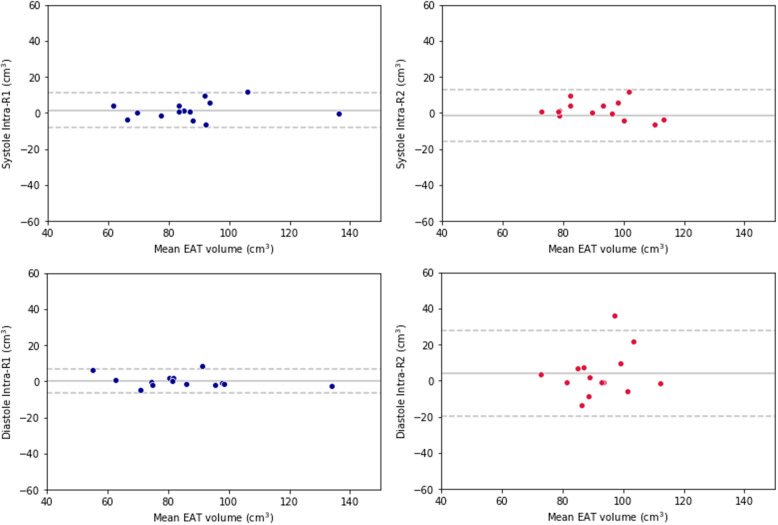
Bland-Altman plots for intra-reader reproducibility of epicardial adipose tissue volume at systole and diastole for the two readers. Dashed lines represent 95% confidence intervals and are placed at ± 2 standard deviations. EAT, Epicardial adipose tissue; R1, Reader 1; R2, Reader 2

R1 displayed the highest intra-reader reproducibility in the diastolic phase, with a bias of 0.3 cm^3^ and a standard deviation (SD) of the differences of 3.3 cm^3^, whereas the lowest intra-reader reproducibility was that of R2 in the diastolic phase, with a bias of 3.9 cm^3^ and an SD of the differences of 12.1 cm^3^. Bland-Altman plots for intra-reader reproducibility are reported in Fig. 2.

**Fig. 3:**
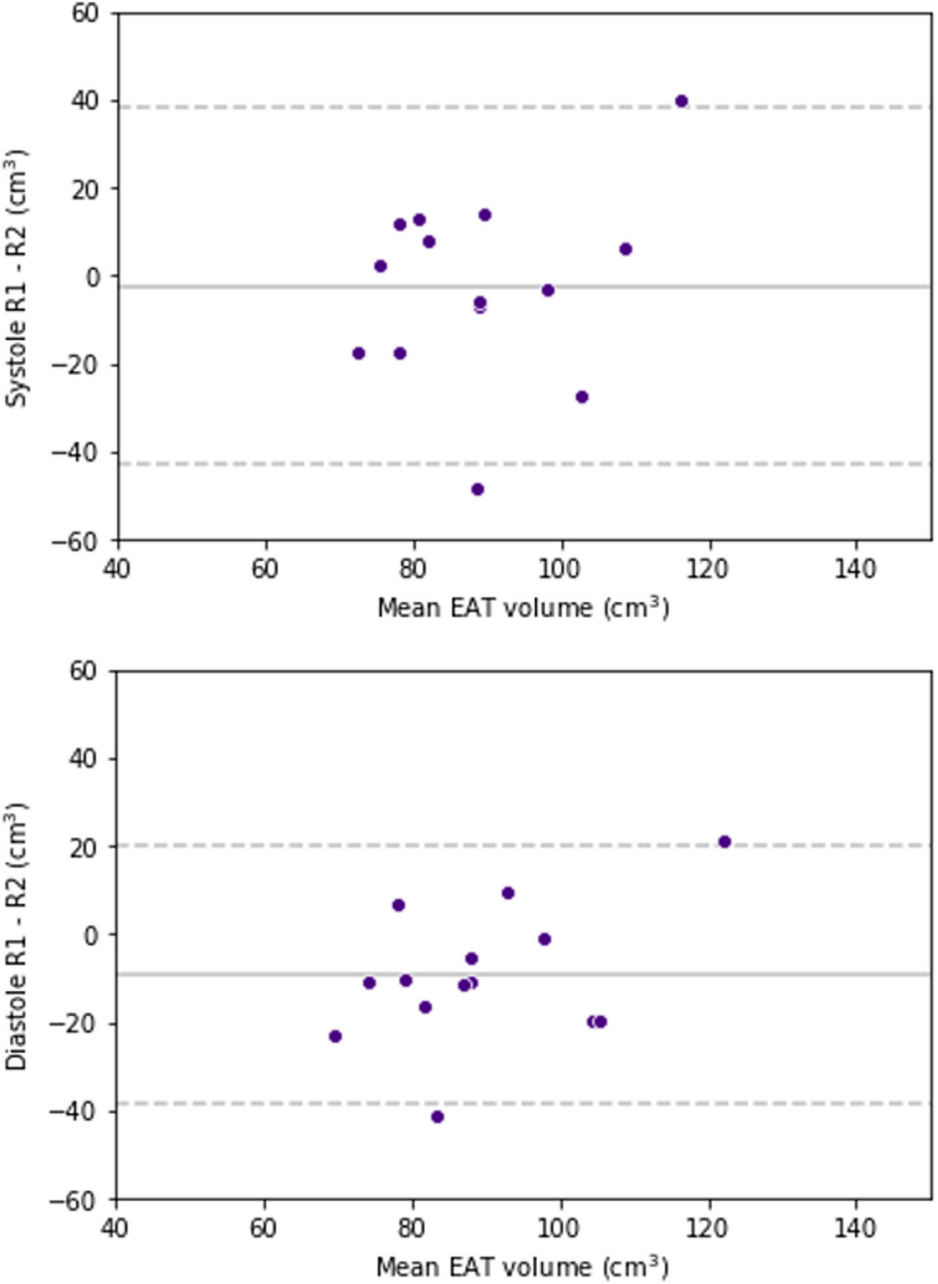
Bland-Altman plots for inter-reader reproducibility of epicardial adipose tissue volume at systole and diastole. Dashed lines represent 95% confidence intervals and are placed at ± 2 standard deviations. EAT, Epicardial adipose tissue; R1, Reader 1; R2, Reader 2

Inter-reader reproducibility was higher in the systolic phase than in the diastolic phase, with a bias of -2.3 cm^3^ and an SD of the differences of 20.6 cm^3^ *versus* a bias of -9.4 cm^3^ and an SD of the differences of 14.9 cm^3^. Bland-Altman plots for inter-reader reproducibility are reported in Fig. 3.

**Fig. 4:**
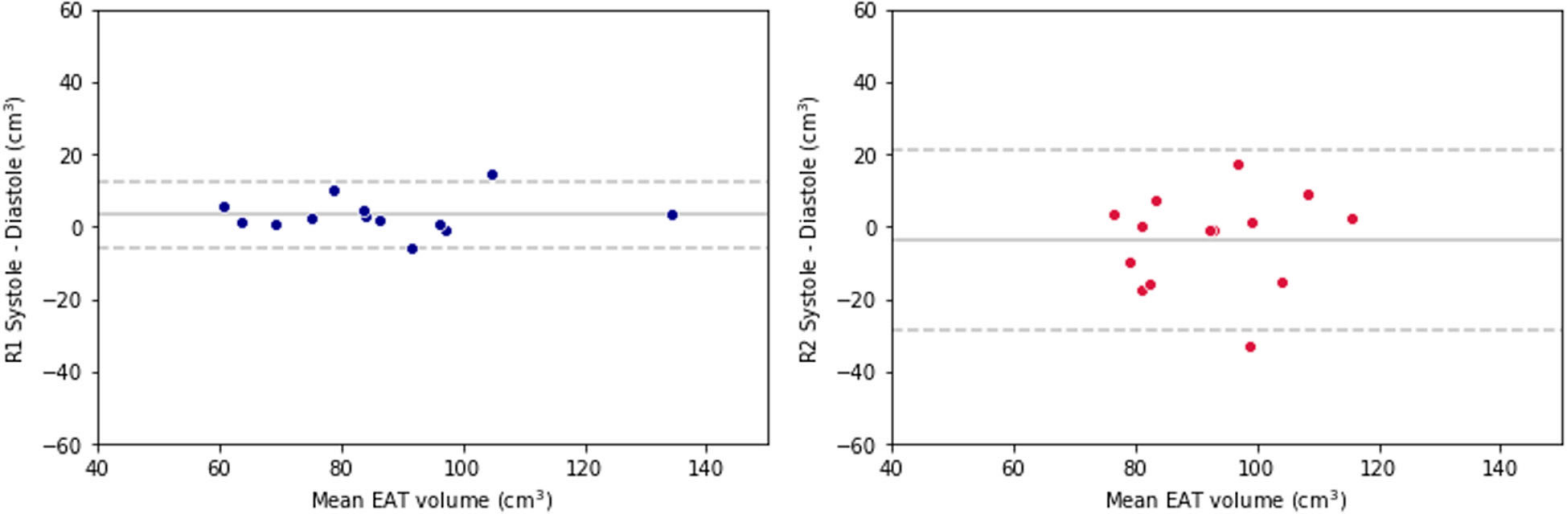
Bland-Altman plots for inter-phase reproducibility between systole and diastole of epicardial adipose tissue volume for the two readers. Dashed lines represent 95% confidence intervals and are placed at ± 2 standard deviations. EAT, Epicardial adipose tissue; R1, Reader 1; R2, Reader 2

Concerning inter-phase reproducibility between systolic and diastolic phases, the magnitude of the bias was similar between R1 and R2, being 3.3 cm^3^ for the former, and -3.8 cm^3^ for the latter, whereas the SD of the differences appeared lower for R1 than R2, being 4.6 cm^3^ for the former and 12.1 cm^3^ for the latter. Systolic EAT volume estimates were significantly greater (*p* = 0.016) than diastolic EAT volume estimates for the first reading by R1, while other sets of measurements did not display significant differences between systole and diastole (*p* ≥ 0.087). Bland-Altman plots for inter-phase reproducibility are reported in Fig. 4.

## Discussion

EAT volume is a well-known biomarker related to cardiovascular risk and disease which can be assessed from volumetric imaging techniques such as computed tomography or MRI without the need for additional scans or contrast agent administration [6].

The average values reported for EAT volumes in our population, with medians ranging from 81 to 93 cm^3^ among different readers and phases, appear within the normal limits for a population with no overt cardiovascular pathology, according to the cut-off of 125 cm^3^ proposed by Spearman et al. [14], in spite of our measurements being performed in an obese population. However, we should note that Shmilovic et al. [15] in a healthy population with low cardiovascular risk showed a higher 95th percentile set at 68.1 cm^3^. This would suggest that patients from our population present with a higher-than-average cardiovascular risk and could be prone to an earlier onset of CAD [16, 17], highlighting the value of EAT volume assessment in an obese population. Therefore, considering the peculiar needs of such patients, namely the necessity of adequately dimensioned MR units, validating the reproducibility of EAT volume assessment on open-bore units could allow a more widespread evaluation of EAT in a population at heightened cardiovascular risk.

Intra-reader reproducibility of EAT volume showed higher precision for the more experienced reader R1, while segmentations repeated by the less experienced reader R2 displayed a high SD implying a wider scattering of volumes around the bias and a minor overall precision. However, the less experienced reader R2 showed a relatively small bias which would most likely not provide clinical impact [18] (the largest being 3.9 cm^3^ at the diastolic phase). Therefore, it may be hypothesised that while segmentations performed by less experienced readers may not yield substantial differences on an average basis, they might not be entirely suitable for single patient assessment due to a higher intrinsic variability.

Concerning inter-reader reproducibility of EAT volume, the higher bias seen in the diastolic phase (-9.4 cm^3^) as compared to the systolic phase (-2.3 cm^3^) may be due to the fact that the segmentation of EAT and pericardium could be easier at systolic phase, as suggested by Malavazos et al. [19], as the contraction of the ventricles leads to EAT appearing thicker and more prominent.

The precision of inter-phase EAT evaluation between systole and diastole appeared to be influenced by reader experience, even though with biases that may suggest a non-substantial clinical impact [18], in spite of a SD of the differences of 12.1 cm^3^ for R2 which could lead to important variation. The significant difference between systole and diastole among values measured by R1 may be due to the intrinsic difference in the prominence of EAT at a ventricular level, which is highlighted by the higher precision and subsequent minor SD of the differences by R1. Such a finding may lead to questioning with regard to the best phase for EAT quantification at cardiac imaging. Nevertheless, previous works have shown that both systolic and diastolic EAT increase with the progression of CAD; therefore, both could be used as biomarkers of pathologic myocardial involvement [20]. Moreover, one additional study by Kang et al. [21] observed a relation between systolic EAT thickness and diabetes, but not with diastolic EAT thickness. This might play in favour of measuring EAT in the systolic phase, due to higher reproducibility and probable clinical impact.

To the best of our knowledge, there is no widespread evidence on the use of open-bore MRI for the assessment of EAT volumes, and thus no available data concerning its reproducibility. The reproducibility of EAT volumes was assessed by previous studies on acquisitions performed on closed-bore MRI systems. For instance, a work by Bettencourt et al. [18] analysing EAT volume reproducibility on 53 patients obtained a bias between 0 and 10 cm^3^, with a SD of the differences which neared 10 cm^3^. Another work by Flüchter et al. [22] performed on 43 patients with congestive heart failure, 24 with CAD and 28 healthy controls displayed an inter-reader bias around 0–5 cm^3^ with a SD of the differences nearing 5 cm^3^. A similar variability was also observed for EAT measurements on computed tomography scans by Commandeur et al. [23], with a bias of 4.35 cm^3^ and a SD of the differences only slightly lower than 10 cm^3^.

Our study presents some limitations. The first is related to its sample size, which is small and only composed of obese patients who met the criteria for bariatric surgery. However, the EAT volumes observed in this population did not differ greatly from those reported for other patients or healthy subjects; therefore, we may hypothesise that reproducibility was not enhanced by the presence of larger EAT volumes. Moreover, the observed reproducibility was comparable to that reported by previous studies [18, 22]. Secondly, a nontrivial number of patients (*n* = 3) were still unable to undergo MRI, even though performed on an open-bore system. Nevertheless, the use of such technology still allowed a considerable number of patients who would not have been able to fit in a traditional, closed-bore unit to undergo MRI imaging. Thus, assessing EAT volumes on scans acquired on open-bore units, despite not being the definitive answer for all obese patients, may provide a solution to increase the number of patients that may be screened for cardiovascular risk by EAT evaluation. Third, we did not include in this work clinical data for correlating EAT volumes and cardiovascular condition. However, we note that the main focus of this work was the evaluation of the precision and reproducibility of EAT volumes quantified on scans acquired on open-bore systems, and once this aim has been completed, the main project will include clinical correlations before and after bariatric surgery.

In conclusion, EAT volume can be quantified on cine acquisitions performed on an open-bore MRI scanner, with a seemingly more precise assessment in the presence of higher reader experience and the use of systolic phase scans. This added feature may prove beneficial, as it could allow the assessment of a subclinical cardiovascular risk biomarker from MRI sequences that are already routinely performed for the evaluation of cardiac function in obese patients. Moreover, as an increasing number of methods for automated segmentation of EAT are being proposed showing promising results also on MRI scans [24], images acquired on open-bore scanners may prove a suitable substrate for future clinical developments.

## Data Availability

The datasets used and/or analysed during the current study are available from the corresponding author on reasonable request.

## Abbreviations

CAD: Coronary artery disease
EAT: Epicardial adipose tissue
IQR: Interquartile range
MRI: Magnetic resonance imaging
R1: Reader 1
R2: Reader 2
SD: Standard deviation
